# GNB5 mutation causes a novel neuropsychiatric disorder featuring attention deficit hyperactivity disorder, severely impaired language development and normal cognition

**DOI:** 10.1186/s13059-016-1061-6

**Published:** 2016-09-27

**Authors:** Hanan E. Shamseldin, Ikuo Masuho, Ahmed Alenizi, Suad Alyamani, Dipak N. Patil, Niema Ibrahim, Kirill A. Martemyanov, Fowzan S. Alkuraya

**Affiliations:** 1 Department of Genetics, King Faisal Specialist Hospital and Research Center, MBC-03, PO Box 3354, Riyadh, 11211 Saudi Arabia; 2 Department of Neuroscience, The Scripps Research Institute, 130 Scripps Way, #3C2, Jupiter, FL 33458 USA; 3 Department of Pediatrics, King Saud Medical City, Riyadh, Saudi Arabia; 4 Department of Neurosciences, King Faisal Specialist Hospital and Research Center, Riyadh, Saudi Arabia; 5 Department of Anatomy and Cell Biology, College of Medicine, Alfaisal University, Riyadh, Saudi Arabia

**Keywords:** Linkage, Mendelian, Neuropsychiatric disorders, Attention deficit hyperactivity disorder (ADHD), G protein coupled receptors (GPCR), Hippocampus, Striatum

## Abstract

**Background:**

Neuropsychiatric disorders are common forms of disability in humans. Despite recent progress in deciphering the genetics of these disorders, their phenotypic complexity continues to be a major challenge. Mendelian neuropsychiatric disorders are rare but their study has the potential to unravel novel mechanisms that are relevant to their complex counterparts.

**Results:**

In an extended consanguineous family, we identified a novel neuropsychiatric phenotype characterized by severe speech impairment, variable expressivity of attention deficit hyperactivity disorder (ADHD), and motor delay. We identified the disease locus through linkage analysis on 15q21.2, and exome sequencing revealed a novel missense variant in *GNB5. GNB5* encodes an atypical β subunit of the heterotrimeric GTP-binding proteins (Gβ5). Gβ5 is enriched in the central nervous system where it forms constitutive complexes with members of the regulator of G protein signaling family of proteins to modulate neurotransmitter signaling that affects a number of neurobehavioral outcomes. Here, we show that the S81L mutant form of Gβ5 has significantly impaired activity in terminating responses that are elicited by dopamine.

**Conclusions:**

We demonstrate that these deficits originate from the impaired expression of the mutant Gβ5 protein, resulting in the decreased ability to stabilize regulator of G protein signaling complexes. Our data suggest that this novel neuropsychiatric phenotype is the human equivalent of *Gnb5* deficiency in mice, which manifest motor deficits and hyperactivity, and highlight a critical role of Gβ5 in normal behavior as well as language and motor development in humans.

**Electronic supplementary material:**

The online version of this article (doi:10.1186/s13059-016-1061-6) contains supplementary material, which is available to authorized users.

## Background

It is increasingly recognized that neuropsychiatric disorders have complex etiology and that many conditions defy classical definitions based purely on phenotypic observations [1]. While there has been tremendous progress towards understanding the genetic basis of hereditary neuropsychiatric conditions, linking specific pathological states to exact molecular alterations has been challenging [2].

One large group of genes with prominent roles in neuropsychiatric disease process encodes components of neurotransmitter signaling cascades acting via G protein coupled receptors (GPCR) [3]. Notably, pharmacological modulation of signaling efficacy at GPCR has been among the most successful strategies for controlling the symptoms of several mental conditions [4]. In the context of neurodevelopmental disorders manifesting in hyperactivity, this frequently includes modulation of signaling via receptors for neurotransmitter dopamine [5]. GPCRs transmit their signals by activating heterotrimeric guanine nucleotide-binding proteins (G proteins). In the basal state, the GDP-bound Gα subunit is tightly bound to the Gβγ heterodimer. Upon successful binding of GPCRs to their ligands, GDP is exchanged for GTP and the heterodimer dissociates such that each of its components can initiate a series of signaling cascades that mediate the net biological effect of the ligand [6].

The strength of the signaling in GPCR cascades is controlled by the members of the regulator of G protein signaling (RGS) proteins, which terminate the signaling initiated by the GPCRs by accelerating the GTP hydrolysis on the Gα subunits, thereby promoting their inactivation. RGS proteins also act as signaling thresholders preventing constitutive and uncontrolled G protein signaling in the absence of GPCR activation [7–11]. In the nervous system, the critical role in controlling GPCR signaling belongs to members of the R7 subfamily of RGS proteins that includes RGS6, RGS7, RGS9, and RGS11. Collectively, R7 RGS proteins have been implicated in learning, motor control, and vision by controlling several neurotransmitter systems including dopamine, opioid, glutamate, and GABA [12]. However, with an exception of the established role of RGS9 in retina pathology [13] and ample evidence from mouse models [14], contributions of R7 RGS proteins to inherited neuropsychiatric conditions in humans has not been documented, despite their strategic role in controlling key relevant processes.

A hallmark of R7 RGS protein organization is their association with Gβ5, a divergent member of the Gβ family through their Gγ-like (GGL) domains [15–17]. Gβ5 is encoded by *GNB5* and shares only ~50 % sequence similarity with classical Gβ1–4 subunits that transmit GPCR signals and are ~90 % identical to each other. Gβ5 also appears to be the only member of Gβ family that can have cellular localization other than the cell membrane, e.g. cytosolic and nuclear [18]. All R7 RGS proteins in vivo exist in complexes with Gβ5, and Gβ5 together with its R7 RGS partners depend on each other for stability; in addition, the GTPase activating protein (GAP) activity of R7 RGS proteins is enhanced several folds when co-expressed with Gβ5 [19–24].

Several lines of evidence support an important neurobiological role of Gβ5. Expression analysis revealed strong enrichment in the brain, particularly in the hippocampus and striatum [25]. More importantly, *Gnb5* knockout mice display a multitude of neurobehavioral abnormalities [26, 27]. Unless assisted for feeding, these mice die shortly after birth. Motor delay persists in the early postnatal developmental period and these mice later develop marked hyperactivity. Interestingly, hyperactivity seen in *Gnb5* deficient mice is paralleled by a number of molecular abnormalities including higher sensitivity of inhibitory GPCR signaling and deficits in basal levels, release, and reuptake of dopamine [28, 29]. Since these disturbed processes have been implicated in the pathogenesis of ADHD in humans, it was suggested that *Gnb5*^–/–^ is a good animal model for this disease [28].

The highly consanguineous nature of the Saudi population provides an ideal setting for the discovery of recessive mutations that are too rare to exist biallelically in outbred populations [30, 31]. We have previously shown this can greatly accelerate the discovery of novel genes for various neurodevelopmental disorders [32, 33]. In this study, we show that an extended consanguineous family reveals the long sought *GNB5*-related phenotype in humans: a neuropsychiatric disorder characterized by severe impairment in acquisition of speech, hyperactivity, attention deficits, and motor delay.

## Methods

### Human participants

All affected family members were evaluated by a certified pediatric neurologist. The diagnosis of ADHD was based on established DSM IV criteria. Intelligence was evaluated using the Wechsler Intelligence Scale for Children (WISC) whenever possible. Affected and available unaffected family members were recruited after signing a written informed consent form as part of an IRB-approved research protocol (KFSHRC RAC#2121053). Venous blood was collected in EDTA and sodium heparin tubes for DNA extraction and the establishment of lymphoblastoid cell lines, respectively. All experimental methods comply with the Helsinki Declaration.

### Autozygosity mapping and linkage analysis

Genome-wide single nucleotide polymorphism (SNP) genotyping was carried out using Axiom SNP Chip Array, which has >500,000 SNPs, following the manufacturer’s instructions (Affymetrix). Determination of the entire set of autozygous intervals per genome (autozygome) used AutoSNPa. We used regions of homozygosity (ROH) >2 Mb in size as surrogates of autozygosity [34]. We then searched for the critical autozygous interval that harbors the disease-causing mutation by comparing the autozygome of affected and unaffected members to identify autozygous intervals that are exclusively shared by the affected members as described before [35]. Linkage analysis was performed using the EasyLINKAGE software. We used a fully penetrant autosomal recessive disease model and assumed homozygosity for the disease-causing mutation based on a shared ancestor.

### Exome sequencing

Exome capture was performed using TruSeq Exome Enrichment kit (Illumina) following the manufacturer’s protocol. Samples were prepared as an Illumina sequencing library, and in the second step, the sequencing libraries were enriched for the desired target using the Illumina Exome Enrichment protocol. The captured libraries were sequenced using an Illumina HiSeq 2500 Sequencer to an average read depth of target regions of 81.8X. The reads were mapped against UCSC hg19 by BWA. The SNVs and indels were detected by SAMTOOLS. WES data were filtered by only considering homozygous variants within the critical autozygous interval, with a MAF <0.001 (as determined by ExAC and 2379 in-house Saudi exomes) [36]. We assessed potential pathogenicity of missense variants based on PolyPhen, SIFT, and CADD.

### Complementary DNA constructs

Plasmid encoding the Flag-tagged, long isoform of the D2 dopamine receptor was a gift from A. Kovoor (University of Rhode Island). pCMV5 plasmids encoding GαoA were gifts from H. Itoh (Nara Institute of Science and Technology, Japan). Plasmids encoding Venus 156-239-Gβ1 and Venus 1-155-Gγ2 were gifts from N. Lambert (Georgia Regents University) [37]. Plasmids encoding RGS9-2, Gβ5, R7BP, and masGRK3ct-Nluc were previously described [38, 39]. Of the two known splice isoforms, we chose to study Gβ5S (NM_006578) for its ubiquitous expression in the nervous system and the lack of the other isoform, Gβ5L, in the brain.

### Real-time monitoring of G protein signaling by fast kinetic bioluminescence resonance energy transfer assay

Agonist-dependent cellular measurements of bioluminescence resonance energy transfer (BRET) between masGRK3ct-Nluc and Venus-tagged Gβγ were performed to visualize the action of G protein signaling in living cells as previously described with slight modifications [39]. HEK293T/17 was transfected with Lipofectamine LTX (12 μL per dish) and PLUS (7.5 μL per 6-cm dish) reagents. Dopamine D2 receptor, GαoA, Venus-156-239-Gβ1, Venus-1-155-Gγ2, masGRK3ct-Nluc, RGS9-2, Gβ5, and R7BP constructs (total 7.5 μg) were used at a 1:2:1:1:1:1:0.5:0.5:0.5 ratio (ratio 1 = 0.42 μg of plasmid DNA). BRET measurements were made with a microplate reader (POLARstar Omega; BMG Labtech) equipped with two emission photomultiplier tubes, allowing us to detect two emissions simultaneously with resolution of 20 ms for every data point. All measurements were performed at room temperature. The BRET signal is determined by calculating the ration of the light emitted by the Venus-Gβ1γ2 (535 nm with a 30-nm band path width) over the light emitted by the masGRK3ct-Nluc (475 nm with a 30-nm band path width). The average baseline value recorded before agonist stimulation was subtracted from BRET signal values and the resulting difference (ΔBRET ratio) was plotted as traces. The rate constant (1/τ) of deactivation phase were obtained by fitting a single exponential function to the traces with Clampfit ver. 10.3 software (Molecular Devices). *k*_GAP_ rate constants were determined by subtracting the basal deactivation rate constant (*k*_app_) from the deactivation rate constant measured in the presence of exogenous RGS9-2/Gβ5 dimer or RGS9-2/Gβ5/R7BP trimer. Obtained *k*_GAP_ rate constants were used to quantify GAP activity of RGS9-2 complexes.

### Immunoblotting

Western blot was carried out to check for the stability of GNB5 protein in patient lymphoblasts compared to control lymphoblasts, using Anti-GNB5(ab185214-Abcam). Briefly, protein was extracted using RIPA buffer (SIGMA) and Halt protease inhibitor cocktail (Thermo-Fisher Scientific), followed by centrifugation at 14,000 *g* at 4 °C for 15 min. Protein obtained in the supernatants was separated by electrophoresis on 4–12 % gradient Tris–glycine gels (Invitrogen) and transferred onto polyvinylidene difluoride membrane (Invitrogen), followed by blocking in 1× PBS with 5 % casein and 0.1 % Tween-20, incubation with primary antibody, and finally incubation with horseradish peroxidase-conjugated IGg secondary antibody. SuperSignal chemiluminescent substrate kit (Pierce) was applied to detect the level of protein expression. Reduction in the protein level was quantified using ImageJ and compared across three independent immunoblots. Immunoblotting of transfected cells were performed as previously reported [22]. To ensure pseudo-linearity of the signal several film exposures were evaluated and non-saturating blots were chosen for the analysis.

## Results

### Identification of a novel autosomal recessive neuropsychiatric disorder

**Fig. 1:**
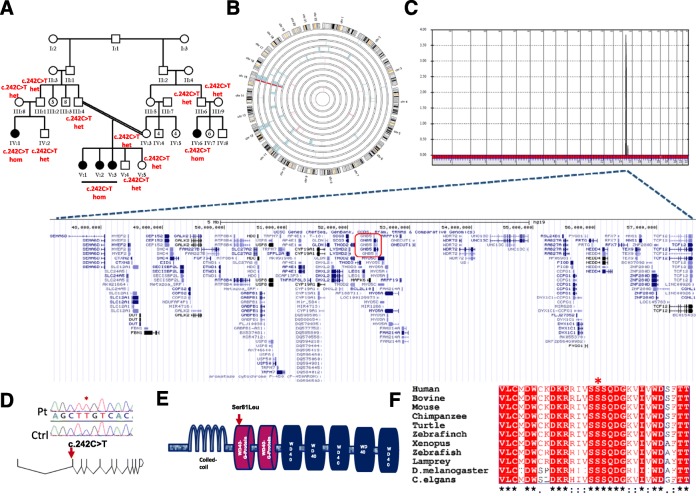
A novel neuropsychiatric disorder is linked to *GNB5* mutation. **a** Pedigree of the study family. **b** *Ideogram* showing a single autozygous interval on chr15 (47,051,884-57,799,765, demarcated by SNPs rs11854077 and rs1280355) that is exclusively shared by the affected members. **c** Genome-wide linkage analysis shows a single linkage peak on chr15 with LOD ~4 that corresponds to the single autozygous interval shown in (**b**). A *screenshot* from the UCSC Genome Browser is shown to highlight the gene content of the linkage peak (*GNB5* is boxed in *red*). **d** *Schematic* of *GNB5* (transcript NM_ 006578) with the sequence chromatogram of the mutation shown on *top*. **e** *Schematic* of Gβ5 and the location of the missense mutation indicated. **f** Strong cross-species conservation of the Ser81 residue denoted with a *red asterisk* (*black asterisks* in the *bottom* denote highly conserved residues)

Through our ongoing effort to identify Mendelian forms of neuropsychiatric disorders in children, we encountered an extended consanguineous Saudi family with multiple members who share the core feature of severe expressive language delay (Additional file 1: Table S1). The five affected members represent three different sibships (Fig. 1). In the first sibship, the index (V:1) is a 10-year-old girl who presented to pediatric neurology with severe expressive and receptive language delay, marked hyperactivity, and school performance issues despite having a normal IQ. She was diagnosed with ADHD according to the DSM IV criteria. Her younger 9-year-old sister (V:2) also had severe expressive and receptive language delay, and although she had no hyperactivity, she met the DSM IV criteria for inattentive type ADHD. Like her sister, she had normal cognitive development. The youngest 3-year-old sister (V:3) was too young to assess for ADHD but, like her other two sisters, had severe language delay. Their first cousin is a 5-year-old girl (IV:1) who initially presented with motor delay and hypotonia but was later found to have severely delayed language development but normal IQ. A distant cousin (IV:6, 9 years old) was not available for formal evaluation but available reports from a different institution showed ADHD diagnosis, severely delayed language acquisition, and mild motor delay.

### A novel neuropsychiatric syndrome is linked to a novel variant in *GNB5*

The pedigree structure was highly suggestive of an autosomal recessive inheritance (the apparent female predominance was likely reflective of the mostly female offspring in the three nuclear families). Therefore, we proceeded with autozygome analysis and identified a single autozygous interval that was exclusively shared by the five affected members IV:1, IV:6, V:1, V:2, and V:3 (Fig. 1). Under the hypothesis that the phenotype observed in this family is caused by homozygosity for a pathogenic mutation within an ancestral haplotype, we proceeded with linkage analysis and the result was fully concordant with autozygosity mapping in that the same critical interval was identified and a significant LOD score of ~4 was obtained (Fig. 1). We then exome sequenced the index and despite full coverage (>10 reads) of all exons of 104 genes in the critical interval, only one novel homozygous variant was identified therein: *GNB5*: NM_006578.3:c.242C > T:p.(S81L). This variant is absent in 2379 Saudi exomes and in the 1000 Genomes, and is present at a very low frequency in ExAC (6 out of 121,000 alleles with MAF of 4.959e-05, 0 homozygotes). It fully segregated with the syndrome in the family such that all affected members were homozygous, parents were heterozygous, while unaffected siblings were either heterozygous or homozygous for the normal allele (Fig. 1). The identified amino acid substitution maps to the first WD40 repeat of the seven-propeller Gβ5 fold within highly conserved region unique to this repeat (Fig. 1d).

### S81L Gβ5 has reduced capacity to deactivate G protein signaling initiated by dopamine receptors

**Fig. 2:**
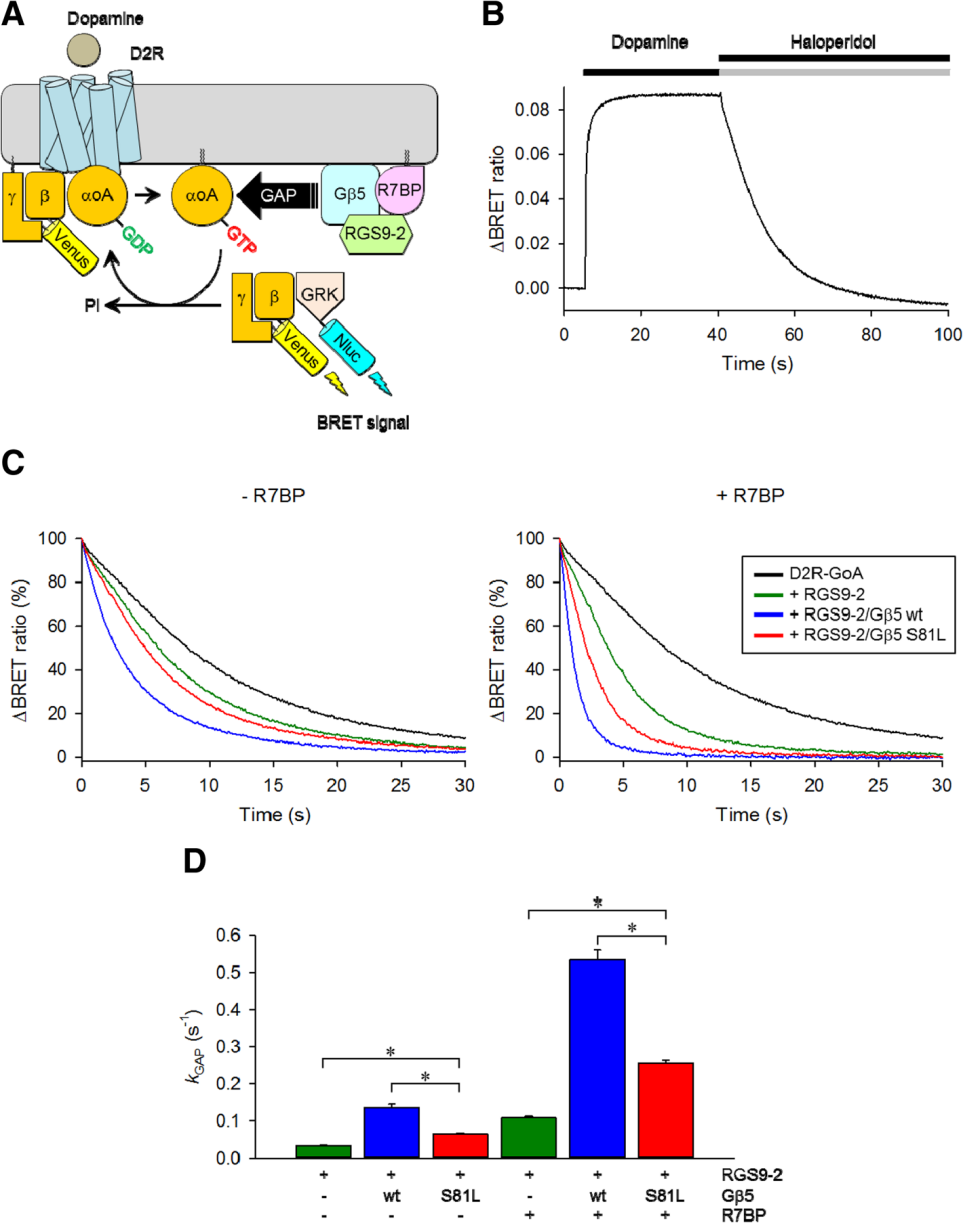
Effect of S81L mutation on GAP activity of RGS9-2 complex. **a** *Schematic* of the assay design. Stimulation of dopamine D2 receptor (D2R) by dopamine results in the dissociation of GαoA from the heterotrimer. Released Venus-tagged Gβγ subunits become available for interaction with Nluc-tagged GRK3ct reporter, producing the BRET signal, which is determined by the change in the emission ratio at 535 nm and 480 nm. RGS9-2/Gβ5 complexes exert GTPase Activating Protein (GAP) activity and accelerate deactivation of G proteins. **b** Representative BRET response of cells reconstituted D2R-GoA signaling. Responses to sequential application of dopamine (100 μM) and haloperidol (100 μM) were recorded. Data are means of six replicates. **c** *Trace lines* represent the deactivation phase of D2R-GoA signaling after haloperidol application to cells transfected with different condition (*left* without R7BP and *right* with R7BP). Data are means of six replicates. **d** *k*_GAP_ rate constants were calculated as an enzymatic activity of RGS9-2 complexes (for further details, see “Methods”) and plotted as a *bar graph*. The same color code was used in panel **c** and **d**. A single *asterisk* (*) indicates *P* <0.0001. One-way ANOVA followed by Dunett’s post-hoc test was conducted with GraphPad Prism Ver. 6. Results shown are representative of two independent experiments each performed with six replicates. Values represent means ± SEM

Given the central role of dopamine in a variety of neuropsychiatric conditions and documented role of R7 RGS-Gβ5 complexes in controlling signaling by the D2 dopamine receptors (D2R) [28], we have next evaluated the functional impact of S81L mutation in Gβ5 on termination of D2R responses, using a representative member of the R7 family, RGS9-2. We used optical means to record activation and deactivation of G protein Go by D2R in living cells by monitoring changes in BRET signal caused by dissociation of Go heterotrimer (Fig. 2a). In this assay, the addition of dopamine resulted in a rapid increase in BRET signal, which returns to the baseline upon the addition of the antagonist haloperidol (Fig. 2b). The speed of this termination phase is accelerated by RGS9, which in turn depends on Gβ5 for its activity (Fig. 2c). Thus, the functional activity of Gβ5 was determined by its ability to speed up D2R deactivation upon the addition of haloperidol in the presence of RGS9-2. Indeed, expressing wild-type Gβ5 substantially accelerated response termination (Fig. 2c, *green* versus *blue* traces in the *left* graph). In contrast, the response offset kinetics was substantially slower in the presence of S81L (Fig. 2c, *blue* versus *red* traces in the *left* graph). Calculating the catalytic efficiency of the reaction by single exponential analysis revealed significantly weaker activity of RGS complexes containing mutant Gβ5 (Fig. 2d). In addition to Gβ5, R7 RGS complexes in vivo contain membrane anchoring subunit R7BP, which further augments their catalytic activity and requires Gβ5for function [38]. Therefore, we next determined the effect of Gβ5 mutation in the presence of R7BP. Again, the addition of WT Gβ5 dramatically facilitated the activity of RGS9-2, but this effect was very modest when S81L Gβ5 was used instead. Therefore, we conclude that S81L results in severe but incomplete loss of function, detrimentally affecting the ability of R7 RGS proteins to deactivate D2R-mediated signaling.

### S81L mutation compromises Gβ5 stability and reduces its ability to augment RGS expression

**Fig. 3:**
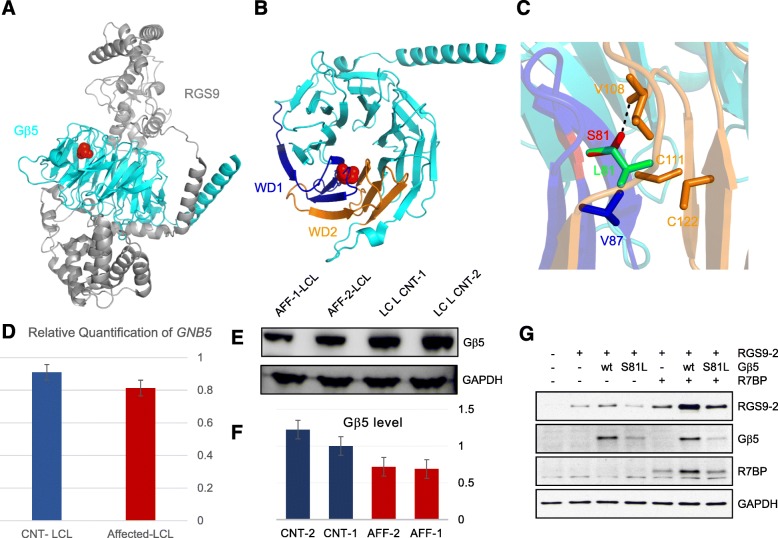
Effect of S81L mutation on protein expression of Gβ5 and RGS9-2 complex. **a** *Cartoon representation* of RGS9-Gβ5 complex crystal structure (PDB ID:2PBI) with S81L mutation shown in the *red sphere*. RGS9 and Gβ5 are shown in *gray* and *cyan*, respectively. **b** *Cartoon representation* of Gβ5 alone (PDB ID:2PBI) with S81 mutation shown in the *red sphere*. Residue S81 is present on β-strand S2β2 of WD1 repeat. WD1 and the neighboring WD2 repeats are represented in *blue* and *orange*, respectively. **c** The hydrogen bond formation of side chain of S81 (*red*) with backbone of V108 (*orange*) is shown as a *dotted black line*. The substituted residue L81 (*green stick*) will not be able to form a hydrogen bond; instead its bulkier side chain will have steric clashes with neighboring amino acids (V87, V108, C111, and C122 represented in stick). All structural representations are made using PyMOL software https://www.pymol.org. **d** Summary of three qRT-PCR experiments (each performed in triplicates) using patient and control LCL to determine the relative abundance of *GNB5* in patient vs. controls showing no significant difference (two-tailed *t*-test p value 0.67). **e**, **f** *Western blot analysis* of GNB5 expression in patient lymphoblastoid cells compared to normal control. **g** *Immunoblot analysis* of protein expression in HEK293T/17 cells. RGS9-2, Gβ5, and R7BP were expressed in different combinations. The proteins extracted from the transfected cells used in BRET assay were subjected to immunoblot analysis using the indicated specific antibodies. Anti-GAPDH antibody was used as a loading control. Representative experiment out of three independent evaluations is shown

We next sought to determine the mechanisms by which S81L mutation affects Gβ5 function. In silico prediction suggests that the S81L variant likely has deleterious structural effects with three algorithms concurring on very high pathogenicity scores (1.0 on PolyPhen, 0.0 on SIFT, and 34 on CADD). To obtain structural insights into the impact of the S81L mutation on Gβ5 at the atomic level, we modeled the consequences of this substitution using crystal structure of RGS9-Gβ5 complex (Fig. 3a, b) [40]. S81 is buried inside the β-strand S2β2 of WD1 repeat close to central axis of β-propeller fold. The S81 is involved in side-chain–main-chain type of hydrogen bond with V108 (Fig. 3c) and such interactions are known to be crucial for maintaining stable structure of the protein [41, 42]. Our modeling suggests that substituting Ser81 with hydrophobic leucine would abolish hydrogen bond formation with V108 and bulkier side chain of leucine at this position would not fit into the tightly packed antiparallel β-sheet of WD1 repeat resulting in a steric clash with neighboring residues (V87 on WD1; V108, C111, and C122 on WD2 (Fig. 3c). Thus, S81L substitution is predicted to compromise Gβ5 folding and/or stability. To test these predictions, we analyzed the expression of Gβ5 in patient-derived lymphoblasts by immunoblotting. Indeed, we detected a modest but consistent reduction of Gβ5 protein levels in the two available lymphoblastoid lines derived from affected patients compared to healthy controls (Fig. 3d, e). In order to rule out the possibility that the apparent reduction in GNB5 protein may have originated at the transcript level, qRT-PCR using patient and control RNA revealed equivalent levels of *GNB5* transcripts (Fig. 3).

To further characterize the effects of the mutation on the expression of Gβ5, we heterologously expressed Gβ5 constructs in HEK293T cells and determined its levels by immunoblotting. Similar to results with lymphoblastoid cells, we observed that S81L Gβ5 mutant had lower expression levels relative to wild-type protein (Fig. 3f). Furthermore, S81L Gβ5 had a reduced capacity to augment the expression of RGS9-2 both in the absence or presence of R7BP, suggesting detrimental effect of the mutation on the folding or stability of the R7 RGS complexes.

## Discussion

The family we present in this study provides a unique opportunity to observe the phenotypic consequence of Gβ5 deficiency in humans. GNB5 knockdown in *C. elegans* results in increased locomotor activity [43]. Knockout of the murine orthologue results in severe hyperactivity and abnormal motor coordination, findings that made us suggest that *GNB5* is a candidate gene for ADHD in humans [28]. ADHD is an extremely common psychiatric disorder that affects 5 % of school-age children, although some surveys estimate the prevalence to be >11 % [44]. Despite its high prevalence and strong heritability, very little is known about its genetics. Like most other complex disorders, information on the genetics of ADHD comes from linkage analysis of families with strong familial aggregation, candidate gene case-control as well as genome-wide association studies (GWAS) [45, 46]. Interestingly, Mendelian forms, which have been identified for many other complex disorders, have not been reported for ADHD to date. Perhaps more surprising is that, unlike other complex disorders, recent advances in sequencing technology have only rarely been exploited in ADHD to identify rare variants that evade detection by traditional GWAS [47].

Motor delay, a consistent feature in *Gnb5* KO mouse, was also variably observed in patients we describe in this study. Similarly, hyperactivity, another prominent phenotype in *Gnb5* KO mouse, was only present at reduced penetrance in the patients we describe with *GNB5* mutation. This could be explained on the basis that these patients have partial whereas *Gnb5* KO mice have complete loss of function. Expectation is that the severity may correlate with severity of Gβ5 disruption and other genetic factors may contribute to how much Gβ5 destabilization the particular mutation would cause, e.g. strength of folding machinery, or lower tone of dopamine signaling in general in unaffected subjects. This suggests that even in this Mendelian form of ADHD, modifiers may play an important role in defining the final phenotype.

Our previously published detailed analysis of the signaling perturbation in the brains of *Gnb5* KO mice suggested a model where increased availability of dopamine is not accompanied by a reciprocal increase in serotonin and that this imbalance may underlie the pathogenesis of hyperactivity in these mice [28]. This model is further supported by our finding that psychostimulant drugs that increased the availability of dopamine failed to treat hyperactivity symptoms whereas drugs that increased the availability of serotonin resulted in a dramatic response [28]. The implication of this on the choice of therapy of *GNB5*-related ADHD remains to be seen.

G protein–gated inwardly rectifying K+ (GIRK/Kir3) channels play an important role in synaptic plasticity and behavior [48, 49]. We have previously shown that Gβ5 co-immunoprecipitates with the GIRK2 and GIRK3 neuronal subunits of GIRK and mediates the formation of GIRK-RGS complex [29]. In hippocampal neurons from *Gnb5*^–/–^ mice, deactivation of GIRK signaling after GABA binding was significantly slower than in the wildtype counterpart. Furthermore, hippocampal CA1 pyramidal neurons from *Gnb5*^–/–^ mice showed altered evoked inhibitory postsynaptic currents, an important inhibitory signaling in the nervous system [29]. These findings are likely relevant to the *GNB5*-related human phenotype because the use of the GABA agonist baclofen was found to induce marked reduction in hyperactivity in *Gnb5*^–/–^ mice [29]. In addition, abnormal synaptic transmission in hippocampal neurons may contribute to the abnormality in expressive language we observe in all five patients with *GNB5* mutation given the role of hippocampal declarative memory system in language development [50]. We also note that mutations in *KCNJ6*, which encode GIRK2, are known to cause a severe neurocognitive phenotype in humans [51]. Finally, it is worth highlighting the role of Gβ5 in regulating adenylate cyclase signaling in the striatum, a major player in motor coordination, as a potential mechanism to explain delayed fine motor coordination observed in patients with *GNB5* mutation [52–54]. Given the obligate nature of Gβ5 association with members of the R7 RGS family and characteristic sensitization of GPCR signaling observed upon Gβ5 elimination, we think that loss of the R7 RGS function underlies the majority of phenotypes in both humans and mice with disabled Gβ5. Consistent with this idea, we report that S81L mutation in Gβ5 detrimentally affects function of a representative member of the R7 RGS family, RGS9-2. Yet we expect that mutations in Gβ5 would similarly affect all members of the R7 family, leading to a global reduction in RGS activity in multiple neuronal circuits across the brain. We should note, however, that at present we cannot rule out the RGS-independent effects associated with the Gβ5 dysfunction, which may be interesting to re-visit if and when additional molecular reactions involving Gβ5 are discovered.

## Conclusions

In conclusion, we suggest that *GNB5* mutation in human results in an autosomal recessive neuropsychiatric disorder that is characterized by severe language delay, fine motor delay, and incompletely penetrant ADHD phenotype. It will be of interest to examine the contribution of rare variants in *GNB5* in future exome/genome sequencing studies of patients with ADHD, especially those with severe speech delay that is out of proportion to their IQ.

## Additional file

Additional file 1: Table S1. Clinical summary of the study patients. Clinical characteristics of the five patients included in this study with homozygous *GNB5* mutation. (DOCX 14 kb)
