# Supplementary material for: GNB5 mutation causes a novel neuropsychiatric disorder featuring attention deficit hyperactivity disorder, severely impaired language development and normal cognition
Source: Genome Biol. 2016 Sep 27;17:195. doi: 10.1186/s13059-016-1061-6 (PMC5037613; doi:10.1186/s13059-016-1061-6)
Supplement: Additional file 1: Table S1. — Clinical summary of the study patients. Clinical characteristics of the five patients included in this study with homozygous GNB5 mutation. (DOCX 14 kb) [file 13059_2016_1061_MOESM1_ESM.docx]

| **ID** | **Age** | **ADHD** | **Speech Delay** | **Motor Delay** | **IQ** | **Facial Dysmorphism** | **OFC** | **MRI** |
| --- | --- | --- | --- | --- | --- | --- | --- | --- |
| **15DG0118** | **10yrs** | **+** | **+** | **-** | 110 | - | 50^th^ centile | Normal |
| **15DG0119** | **9yrs** | **+** | **+** | **-** | Not done* | - | 50^th^ centile | Not done |
| **15DG0120** | **3yrs** | **? (too young)** | **+** | **+** | Not done* | - | ? | Not done |
| **15DG1623** | **5yrs** | **-** | **+** | **+** | 80 | - | 50^th^ centile | Normal |
| **16DG0232** | **9yrs** | **+** | **+** | **+** | ? | - | ? | Normal |

*Although no formal IQ testing was performed, the clinical assessment was consistent with normal intelligence.
